# Is suture cerclage fixation a valid treatment for intraoperative nondisplaced calcar fractures in reverse total shoulder arthroplasties?

**DOI:** 10.1016/j.jseint.2021.03.008

**Published:** 2021-04-24

**Authors:** Philipp Kriechling, Anita Hasler, Caroline Passaplan, Karl Wieser

**Affiliations:** Department of Orthopedics, Balgrist University Hospital, Zürich, Switzerland

**Keywords:** Reverse total shoulder arthroplasty, Complication, Intraoperative humeral shaft fracture, Suture cerclage fixation, FiberWire

## Abstract

**Background:**

Implantation rates of reverse total shoulder arthroplasties continue to grow worldwide. Despite satisfying results, a distinct number of complications persist. Intraoperative fractures of the humeral shaft might occur in a certain number of cases. The literature is sparse regarding incidence and treatment options. This study analyzed the treatment using suture cerclage or stainless-steel-cable cerclage.

**Methods:**

Our prospectively followed-up cohort of 860 patients who received primary reverse total shoulder arthroplasty in a tertiary referral hospital between September 2005 and August 2018 was screened for intraoperative medial humeral calcar fractures. The patients were retrospectively analyzed as per the treatment algorithm using (1) suture cerclage with FiberWire, (2) cable cerclage with stainless steel cable, or (3) no intervention. The outcome was radiologically and clinically (Subjective Shoulder Value and Constant score) evaluated.

**Results:**

A total of 39 (4.5%) intraoperative calcar fractures of the humeral shaft were identified with 29 cases available for analysis at a mean follow-up time of 52 ± 27 months. Sixteen of them were treated with suture cerclage, 7 with metal cable cerclage, and 6 without intervention. All fractures were nondisplaced or could be reduced anatomically and healed without any stem subsidence or loosening within the first 4.5 months. The intervention groups reached similar values for the Subjective Shoulder Value (68%±27% vs. 79%±19%, suture vs. cable) and relative Constant score (65 % ± 25 % vs. 75 % ± 23).

**Conclusion:**

Intraoperative medial calcar fractures can be sufficiently treated with metal or suture cerclage fixation. High-strength polyblend-polyethylene sutures seem to be a valid therapeutic option for selected medial calcar fractures of the humerus. In selected cases, however, benign neglect can result in excellent results as well.

The implantation rates of reverse total shoulder arthroplasties (RTSAs) continue to grow owing to an increasing number of indications^11^ as well as an aging society.^16^ The original indication for Grammont’s RTSA was cuff tear arthropathy.^13^ Nowadays, the indications include irreparable rotator cuff tear, primary eccentric osteoarthritis, acute or chronic fracture treatment, and revision surgeries. A higher total amount of complications accompanies the increasing number of indications and consecutively implanted RTSAs.^3^ Zumstein et al^24^ reported in a meta-analysis a total complication rate of 24%. Among them, intraoperative humeral fractures are described in up to 16% of the complications. ^24 21^ Reasons might be osteoporotic bone in an aging society, anatomic differences of the humeral shaft, predetermined lesions owing to an underlying fracture, or different implantation techniques.^9^ The optimal intraoperative treatment is sparsely investigated in the existing literature.^7^^,^^8^^,^^17^^,^^22^ Current concepts include treatment with sutures cerclages, stainless-steel cerclages, or simply nothing.^9^ Renner et al^17^ compared medial calcar fracture stabilization using suture cerclage or metal cable cerclage in a cadaveric study. They reported similar fixation strength between the 2 techniques for nondisplaced fractures.

Except the few existing studies on intraoperative humeral shaft fractures, no studies investigated the outcome in isolated medial calcar fractures so far.

The aim of this study was, therefore, to analyze the outcome of intraoperative, medial humeral shaft fractures during RTSA implantation using suture cerclage (FiberWire), stainless-steel-cable cerclage, or no fixation. We hypothesized that the fixation method had no influence on the clinical and radiological outcome.

## Material and methods

### Ethics approval

This study was approved by the Ethics Committee of the University of Zürich (ID 2018- 01494) and conducted following the Helsinki Declaration.

### Patients selection

A total of 1196 RTSAs were implanted in our tertiary referral hospital between September 2005 and August 2018, 860 (72%) cases of them were primary arthroplasties. Inclusion criteria were medial calcar fracture as per the surgical report and/or conventional postoperative x-rays, patients older than 18 years, primary reverse shoulder arthroplasty, complete clinical and radiological follow-up of minimum 2 years, and signed informed consent. Intraoperative medial calcar fractures were defined as all displaced or nondisplaced fractures of the medial proximal humerus extending or not extending into the meta-diaphyseal region. Isolated fractures of the tuberosities were excluded.

### Clinical and radiological examination

The patients were followed up regularly at 6 weeks, 18 weeks, and 2-4 years postoperatively. Each consultation included standardized conventional radiographic examination (anteroposterior and axillary lateral view) and clinical evaluation using the Subjective Shoulder Value^12^ and Constant-Murley score,^6^^,^^12^ including measurement of the abduction force with a validated dynamometer (Isobex; Cursor, Bern, Switzerland). All the patients underwent standardized preoperative and postoperative clinical evaluation by one staff medical study nurse under an orthopedic surgeon's supervision specialized in orthopedic shoulder treatment. Radiological evaluation was performed by one author of the study (PK), assessing time to fracture union, stem subsidence, and stem loosening. All available radiographs were analyzed. (Maximum follow-up is shown in Table I)

**Table I:** Displays the demographic data of the suture cerclage group (SC), the cable cerclage group (CC), and the group without intervention.

	Suture (n = 16)	Cable (n = 7)	Nothing (n = 6)	*P* value (SC vs. CC)
Follow-up (m)	32 ± 15	74 ± 15	73 ± 14	0.01
Gender				
Female	11 (69%)	5 (71%)	4 (67%)	0.90
Male	5 (31%)	2 (29%)	2 (33%)	
Age at surgery (y)	71 ± 9	70 ± 10	67 ± 5	0.74
Body mass index	28 ± 6	25 ± 1	25 ± 7	0.18
Height (cm)	160 ± 10	167 ± 8	167 ± 8	0.28
Weight (kg)	74 ± 20	70 ± 8	68 ± 14	0.87
ASA				
ASA 1	1	1	0	0.87
ASA 2	8	3	4	
ASA 3	7	3	2	

### Surgical technique

**Figure 1:**
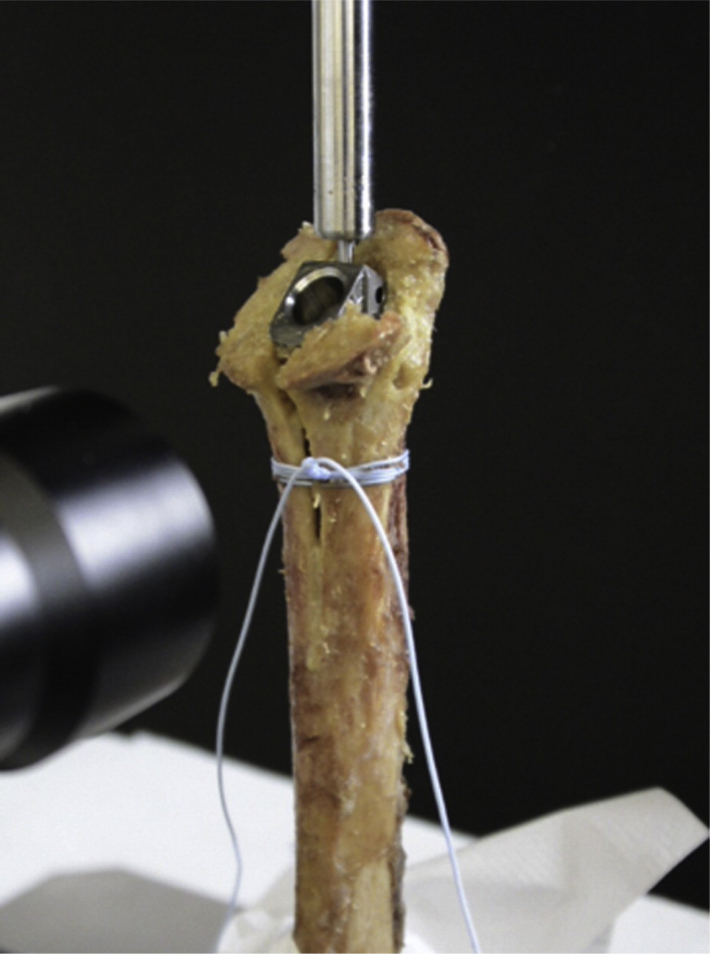
Suture cerclage fixation using FiberWire 5.0.

The total joint replacement was performed by 11 fellowship-trained staff shoulder surgeons in a standardized manner. Antibiotic prophylaxis with Cefuroxim 1.5 g (Fresenius Kabi, Switzerland) was administered intravenously 30 minutes before skin incision. The patient was placed in a beach chair position with general or regional anesthesia. Disinfection with Betaseptic (Mundipharma Medical Company, Switzerland) and draping was performed with 3 rectangular drapes, 2 u-shaped drapes, and an adhesive incisional drape (Ioban; 3M, USA) in all the patients. A deltopectoral approach was used in all cases, leaving the cephalic vein laterally. The humeral head was resected, and the humeral stem was prepared to fit the planned implant size. The stem was inserted in 0° to 20° of retroversion. A press-fit implantation technique was chosen in case of good fixation. Cemented stem implantation was performed in cases of poor implant fixation where a press-fit stem fixation was impossible. Of the 19 that were cemented, 10 cases (42%), 4 cases (44%), 4 cases (57 %) were cemented in the suture cerclage, metal cable cerclage, and no-intervention groups, respectively. The treatment of the medial calcar fractures with suture cerclage or cable cerclage was decided by the operating surgeon. The timing of the cerclage was dependent on the occurrence of the fissure. In the case of calcar fractures during broaching, cerclage was performed before insertion of the definitive stem; in the case of fractures after insertion of the definitive implant, cerclage was performed afterward. For suture cerclage fixation, No. 5 FiberWire (Arthrex, Naples, FL, USA) was used 2 to 6 times. (Fig. 1) The metal cerclage was passed using a shuttling device and tightened with pliers.

Picture with reprint permission Elsevier (License 4960740921461) from Renner N et al (https://doi.org/10.1016/j.jse.2014.02.012).

The glenoid was reamed to create a flat surface. Subchondral bone was only removed if it prevented stable positioning of the prosthetic component with the baseplate flush with the inferior glenoid rim. The baseplate was implanted with a neutral version and neutral to slight inferior inclination not exceeding 10°. All the patients received a Zimmer Anatomical Shoulder Inverse/Reverse with a standard shaft except 2 patients in the metal cerclage group who received a fracture shaft for the surgical indication of proximal humeral fractures, one cemented and one uncemented. If possible, a transosseous subscapularis refixation using No. 2 FiberWire was carried out. Aftercare consisted of wearing a sling for 6 weeks allowing passive mobilization and minimal active use of the arm. Active range-of-motion exercises were carried out without weight through weeks 7 to 12.

### Statistical analyses

Study data were collected and managed using Research Electronic Data Capture, version 8.6, hosted at Balgrist University Hospital.^14^^,^^15^

The statistical analyses were performed using SPSS software v24.0 (IBM, Armonk, NY, USA). The normal distribution of variables was tested with the Shapiro-Wilk test and compared preoperative and postoperative scores with the paired t-test (parametric data) or the Wilcoxon rank sum test (nonparametric distribution). Fisher's exact test was used for categorical variables. A *P* value less than 0.05 was considered significant. Owing to the given population of patients with medial calcar fractures, no power analysis was carried out.

## Results

A total of 39 patients could be identified with intraoperative, medial calcar fractures. Twenty-three patients were treated with suture cerclage (59%), 9 patients were treated with metal cerclages (23%), and 7 patients (18%) received no specific fracture treatment. In the suture cerclage group, the fracture occurred in 10 patients during broaching, in 12 patients during impaction, and in 1 case, it was not clearly described. Cementation was used in 7 of 10 cases with fracture after broaching and in 3 of 12 cases with fracture due to impaction. In the cable cerclage group, the fracture occurred during broaching in 4 cases, during impaction in 4 cases, and was undescribed in 1 case. Cementation was used in 3 of 4 cases with fracture after broaching and 0 of 4 cases with fracture during impaction. The 7 patients with no intervention need further description because in 2 of the cases, the fracture was not recognized intraoperatively and, therefore, not treated. The implant position was rated as very stable intraoperatively. In 2 patients, a very thin and short fracture occurred during broaching of the stem. The intraoperative assessment showed a stable implant without the need for additional fixation. Another fracture was rated stable, as it occurred during impaction of the stem. Two patients were treated using shaft cementation owing to small calcar fractures and reached stable impaction. The 5 recognized and the 2 unrecognized fractures were described in the surgical report as very stable without further need for cerclage fixation.

**Figure 2:**
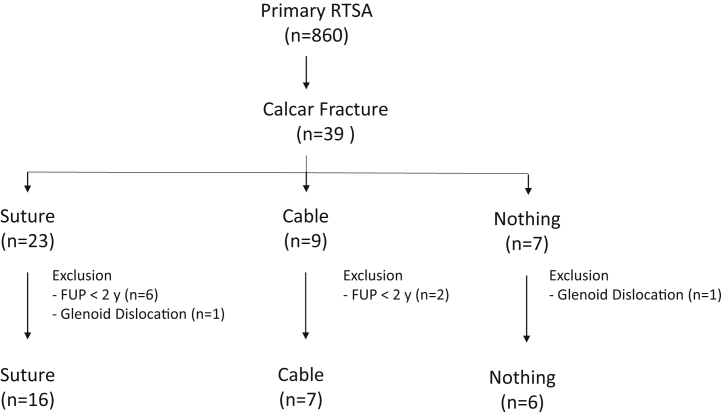
Flowchart presenting the included patients. *Cable,* stainless-steel-cable cerclage fixation; *FUP*, follow-up; *Nothing*, no cerclage fixation; *RTSA*, reverse total shoulder arthroplasty; *Suture*, suture cerclage using FiberWire.

Applying the inclusion criteria, 10 patients had to be excluded: in 8 cases because of follow-up less than 2 years and in 2 cases because of revision surgery with conversion to hemiarthroplasty. All were unrelated to the intraoperative fracture. (Fig. 2) Demographics of the analyzed patients are listed in Table I.

### Clinical outcome

The postoperative comparison among the suture cerclage group, the metal cable cerclage group, and the group without intervention did not reveal statistically significant differences. All 3 groups achieved similar results in the Constant score, Subjective Shoulder Value, pain, range of motion, and abduction strength. For the suture cerclage fixation group, the time point of the latest follow-up was used. To increase comparability, follow-up after 2 or 3 years was chosen for statistical analysis of outcome in the cable cerclage group and the group without intervention. (Table II) A comparison of all latest follow-ups also showed no statistical differences between groups. (Supplemental Table 1) Preoperative to postoperative comparison revealed improvement in the Constant score and Subjective Shoulder Value for all 3 groups. (Table III and supplemental Table 2)

**Table II:** The clinical outcome parameters between the 3 groups preoperatively and postoperatively.

Variable	Suture (SC)		Cable (CC)		Nothing (N)		*P* value∗
	Preoperatively	Postoperatively	Preoperatively	Postoperatively	Preoperatively	Postoperatively	
Number	N = 13	N = 16	N = 7	N = 7	N = 6	N = 6	
Absolute CS	31 ± 16 (11; 70)	54 ± 21 (23; 81)	26 ± 15 (5; 49)	63 ± 21 (26; 82)	29 ± 11 (18; 47)	65 ± 12 (45; 78)	0.44, 0.37, 0.85
Relative CS (%)	39 ± 21 (13; 84)	65 ± 25 (25; 101)	33 ± 17 (7; 59)	75 ± 23 (32; 96)	36 ± 14 (23; 58)	77 ± 11 (58; 90)	0.46, 0.44, 0.85
SSV (%)	34 ± 11 (20; 50)	68 ± 27 (20; 100)	44 ± 28 (10; 80)	79 ± 19 (45; 100)	24 ± 20 (0; 60)	81 ± 13 (55; 92)	0.44, 0.52, 0.9
CS pain *(0 to 15, 15 best)*	6 ± 3 (1; 12)	14 ± 2 (9; 15)	4 ± 2 (1; 8)	14 ± 2 (11; 15)	6 ± 5 (0; 13)	11 ± 4 (7; 15)	0.44, 0.05, 0.25
Flexion (°)	73 ± 42 (20; 160)	99 ± 38 (20; 150)	70 ± 35 (10; 100)	114 ± 43 (50; 150)	74 ± 46 (20; 160)	126 ± 17 (110; 160)	0.28, 0.12, 0.95
Abduction (°)	65 ± 39 (20; 160)	102 ± 42 (30; 160)	73 ± 35 (20; 110)	120 ± 47 (50; 165)	61 ± 46 (30; 160)	126 ± 21 (100; 160)	0.48, 0.31, 0.85
External rotation (°)	28 ± 25 (-10; 70)	22 ± 26 (-20; 65)	15 ± 35 (-30; 70)	32 ± 20 (0; 60)	51 ± 24 (10; 90)	34 ± 22 (0; 70)	0.4, 0.33, 0.95
Internal rotation *(0 to 10, 10 best)*	4 ± 3 (0; 10)	5 ± 3 (0; 8)	5 ± 3 (0; 10)	6 ± 3 (2; 10)	4 ± 3 (0; 6)	6 ± 3 (2; 10)	0.73, 0.63, 0.9
Abduction strength (kg)	1 ± 2 (0; 5)	2 ± 2 (0; 6)	0 ± 1 (0; 2)	3 ± 2 (0; 6)	1 ± 1 (0; 3)	3 ± 2 (0; 5)	0.54, 0.54, 1
Follow-up (mo)		32 ± 15 (24; 66)		29 ± 6 (24; 36)		34 ± 13 (24; 60)	0.21, 0.16, 0.85

*CC*, cable cerclage; *CS*, Constant score; *N*, no intervention; *SC*, suture cerclage; *SSV*, subjective shoulder value.

*P* values display the statistical significant difference between all the groups.

∗ SC vs. CC, SC vs. N, CS vs. N.

**Table III:** Comparison between preoperative and postoperative *P* values for all 3 groups.

	Suture	Cable	Nothing
Absolute CS	0.00	0.01	0.00
Relative CS	0.00	0.01	0.00
SSV	0.00	0.05	0.00
CS pain	0.00	0.00	0.05
Flexion	0.01	0.04	0.00
Abduction	0.00	0.07	0.00
External rotation	0.97	0.18	0.00
Internal rotation	0.08	0.86	0.14
Force	0.04	0.05	0.02
Follow-up (mo)	32 ± 15	29 ± 6	34 ± 13

*CS*, Constant score; *SSV*, Subjective Shoulder Value.

### Radiological outcome

All intraoperative medial humeral shaft fractures could be unioned using suture cerclage, metal cable cerclage, or no intervention. In the further course, no fractures did secondarily displace, and all healed within the first 4.5 months. No stem subsidence or loosening was seen until the latest follow-up.

### Complications

A total of 6 complications occurred (15%) with revision surgery in 4 patients (10%).

Two patients in the suture cerclage group had an acromion fracture at 5 and 14 months postoperatively, 1 related to a fall. Both healed without surgical intervention. One patient in the suture cerclage group underwent revision surgery by greater tuberosity refixation and a second intervention for consecutive infection with debridement and component change. In the further course, the infection healed. Another patient suffered from glenoid dislocation at 25 months postoperatively with need of revision surgery and conversion to hemiarthroplasty.

In the cable cerclage group occurred a case of instability with shoulder dislocation, which became stable after revision surgery.

The group without cerclage intervention included 1 patient with glenoid dislocation at 84 months postoperatively with revision surgery and conversion to hemiarthroplasty.

## Discussion

The most important finding of this study was that intraoperative medial calcar fractures occurred in 4.5% of our patients with primary RTSA using a medial metaphyseal-engaging stem. All of them could be intraoperatively stabilized with suture cerclage, cable cerclage, or in selected cases without any other fixation device. All fractures were considered healed after a mean of 4.5 months without any stem subsidence or loosening up to the final radiographic follow-up. There was furthermore no difference between all clinical outcome parameters at a standardized follow-up period between the groups.

Intraoperative complications occur in a certain number of RTSAs. Zumstein et al^24^ reported an incidence of 2% for intraoperative humeral fractures. Some risk factors for the occurrence of intraoperative fractures are known. Stem design and implantation techniques play a crucial role. Athwal et al^1^ described a press-fit stem design for anatomic total shoulder arthroplasties and Singh et al^19^ described female gender and previous shoulder instability as risk factors. Another risk factor is osteonecrosis ^4^ and, as described in the following text, revision surgery.^1^^,^^21^ Only a few studies investigated intraoperatively treated humeral fractures with suture cerclages, with no one analyzing explicitly medial calcar fractures.^4^^,^^9^

Eyberg et al^8^ recently published the suture cerclage–only treatment of 12 humeral shaft fractures and 15 osteotomies in 27 cases (3 primary RTSAs, 24 revision RTSAs) with a follow-up of 12.6 months (0.8 to 42 months). They reported bone healing in all shoulders with satisfying clinical outcome measures. Albeit, no conclusion for other fixations methods can be made. Furthermore, all humeral fractures were included instead of calcar fractures only.

Garcia-Fernandez et al^10^ reported in 2015 three intraoperative humeral fractures (1.5%) in their cohort of 203 patients with a follow-up of 79 (12 to 141) months. Contrary to the study by Eyberg et al,^8^ they were treated with steel cerclages and reported satisfying clinical outcomes and complete bone healing at the latest follow-up.

Atoun et al^2^ reported on 2 intraoperative fractures in 31 (6.5%) patients who received a short-stemmed RTSA. Both fractures were treated conservatively without any fixation, both fractures healed.

Wagner et al^21^ reported RTSA and identified 32 (16%) intraoperative humeral fractures in a prospectively followed up cohort of 230 revision shoulders arthroplasties. The fractures were mainly related to implant removal in 81% of the cases, and associated with canal preparation and impaction in 19%. The fractures were stabilized with additional fixation in 8 of the case. Four of them occurred to the metaphysis, which received metal sutures and 4 occurred to the tuberosities, which received suture cerclage fixation. No further analysis between the suture techniques has been reported. All patients reached similar results compared with patients without intraoperative fracture with more than 90% reporting pain relief.

Most data are available on total anatomic shoulder arthroplasty or hemiarthroplasties. Singh et al^19^ studied the frequency of periprosthetic fractures in primary anatomic shoulder arthroplasties from a prospectively followed up cohort at the Mayo Clinic. They identified intraoperative humeral fractures in 48 of 4019 cases (1.2%); of which, 28 (0.7%) were defined as proximal humeral fractures. The incidence was similar to previously published data of the same group.^1^^,^^5^ Singh et al^19^ reported that no special treatment was necessary for most of the 28 proximal, intraoperative fractures. They used additional sutures in 48% of proximal fractures in anatomic total shoulder arthroplasties. Unfortunately, the influence on the clinical outcome was not reported.

We recorded a total of 6 complications (15%) in our cohort, none of them related to the fixation method. Eyberg et al^8^ published similar data with a complication rate of 11% (3 shoulders, 1 periprosthetic fracture unrelated to the intraoperative one, and 2 periprosthetic infections); of which, all underwent revision surgery. Wagner et al^21^ reported 2 postoperative, conservatively treated fractures (0.9%) with a total complication rate of 2.2%. Revision surgery was necessary in 3 patients (1.3%), possibly all unrelated to the intraoperative fracture (1 instability and 2 glenoid loosenings).

Our study showed similar results for suture cerclage fixation and cable cerclage fixation in medial calcar fractures. At the beginning of the observational period, cable cerclages were used, and satisfying fracture healing was achieved. However, metal cerclages can theoretically reduce the periosteal blood flow,^23^ might interfere with the radiographic imaging, and might break during the postoperative course, potentially leading to wear and metallosis.^18^ Renner et al^17^ showed in a cadaveric study similar fracture stabilization in nondisplaced fractures using high-strength polyblend-polyethylene suture fiber fixation with cow-hitch knot fixation (Fig. 1) compared with stainless-steel-metal cerclages. They tested tightening force, load to 3-mm gap and load to total failures as well as humeral stem subsidence while implantation in a fractured cadaveric shaft. After these encouraging biomechanical results, we started to use this technique in clinics as standard treatment in case of potential unstable medial calcar fractures.

No treatment algorithm on intraoperative calcar fractures exists so far. For humeral fractures in general, it was recommended to replace the shaft for a longer and/or bigger one, if technically achievable. The change for a cemented stem might also be an option. For more proximal humeral fractures, treatment with sutures and metal cerclages alone might be enough.^9^^,^^20^

Several limitations in our study have to be mentioned. (1) The data were collected retrospectively from our prospectively followed up RTSA cohort. (2) Different surgeons made the intraoperative treatment decision without a definite treatment algorithm owing to missing data in the literature. (3) Humeral stem design possibly might affect the occurrence of intraoperative fractures. Because this is a monocentric study and all patients received the same medial metaphyseal-engaging implant, no association can be made to the implant design. (4) This study could show similar calcar fracture treatment results for suture cerclage fixation and cable cerclage fixation, which could lead to a change in intraoperative fracture treatment. (5) Albeit, conservative treatment without any cerclage fixation showed promising results as well. That inevitably raises the question if intraoperative calcar fracture treatment is necessary at all and at which indication. However, owing to the retrospective study design, a strong selection bias cannot be excluded. It is very likely that the patients without intervention showed only small and superficial fractures without causing instability of the stem and received, therefore, no cerclage.

Therefore, further investigation is mandatory to determine in which cases of calcar fracture a suture or cable cerclage fixation is necessary.

## Conclusion

Intraoperative medial calcar fractures can be sufficiently treated with metal or suture cerclage fixation. High-strength polyblend-polyethylene sutures seem to be a valid therapeutic option for selected medial calcar fractures of the humerus. In selected cases, however, benign neglect can result in excellent results as well.

## Disclaimers:

*Funding:* No funding was disclosed by the authors.

*Conflicts of interest:* The authors, their immediate families, and any research foundations with which they are affiliated have not received any financial payments or other benefits from any commercial entity related to the subject of this article.
