# Supplementary material for: Is suture cerclage fixation a valid treatment for intraoperative nondisplaced calcar fractures in reverse total shoulder arthroplasties?
Source: JSES Int. 2021 Apr 24;5(4):673–8. doi: 10.1016/j.jseint.2021.03.008 (PMC8245986; doi:10.1016/j.jseint.2021.03.008)
Supplement: Supplemental Table 1 [file mmc1.docx]

Supplemental table 1: Displays the Clinical Outcome Parameters preoperatively and postoperatively between the three groups at the latest follow-up. P-Values displays the statistical significant difference between all the groups. * SC vs. CC, SC vs. N, CS vs. N. Abbreviations: CC = Cable Cerclage, CS = Constant Score, N = No intervention, SC = Suture Cerclage, SSV = Subjective Shoulder Value

| **Variable** | **Suture (SC)** | | **Cable (CC)** | | **Nothing (N)** | | **p-Value *** |
| --- | --- | --- | --- | --- | --- | --- | --- |
|  | Preop | Postop | Preop | Postop | Preop | Postop |  |
| **Number** | N=13 | N=16 | N=7 | N=7 | N=6 | N=6 |  |
| **Absolute CS** | 31 +- 16 | 54 +- 21 | 26 +- 15 | 59 +- 24 | 30 +- 11 | 63 +- 9 | 0.82, 0.61, 0.94 |
| **Relative CS (%)** | 39 +- 21 | 65 +- 25 | 33 +- 17 | 73 +- 24 | 38 +- 14 | 76 +- 10 | 0.76, 0.62, 0.97 |
| **SSV (%)** | 34 +- 11 | 68 +- 27 | 44 +- 28 | 72 +- 29 | 24 +- 22 | 84 +- 13 | 0.94, 0.40, 0.67 |
| **CS Pain**  ***(0 to 15, 15 best)*** | 6 +- 3 | 14 +- 2 | 4 +- 2 | 13 +- 4 | 6 +- 5 | 13 +- 4 | 0.67, 0.82, 0.98 |
| **Flexion (°)** | 73 +- 42 | 99 +- 38 | 70 +- 35 | 110 +- 47 | 73 +- 51 | 114 +- 22 | 0.79, 0.67, 0.98 |
| **Abduction (°)** | 65 +- 39 | 102 +- 42 | 73 +- 35 | 120 +- 55 | 65 +- 49 | 120 +- 37 | 0.65, 0.68, 1.00 |
| **External Rotation (°)** | 28 +- 25 | 22 +- 26 | 15 +- 35 | 19 +- 28 | 58 +- 18 | 39 +- 10 | 0.98, 0.30, 0.32 |
| **Internal Rotation**  ***(0 to 10, 10 best)*** | 4 +- 3 | 5 +- 3 | 5 +- 3 | 5 +- 3 | 3 +- 3 | 5 +- 3 | 0.88, 0.92, 1.00 |
| **Abduction strength (kg)** | 1 +- 2 | 2 +- 2 | 0 +- 1 | 2 +- 2 | 1 +- 1 | 2 +- 2 | 0.69, 0.95, 0.91 |
| **Follow-up (months)** |  | 32 +- 15 |  | 74 +- 15 |  | 73 +- 14 |  |
