# Supplementary material for: Is suture cerclage fixation a valid treatment for intraoperative nondisplaced calcar fractures in reverse total shoulder arthroplasties?
Source: JSES Int. 2021 Apr 24;5(4):673–8. doi: 10.1016/j.jseint.2021.03.008 (PMC8245986; doi:10.1016/j.jseint.2021.03.008)
Supplement: Supplemental Table 2 [file mmc2.docx]

Supplemental table 2: Comparison between preoperative and latest postoperative follow-up for alle three groups. Abbreviations: CS = Constant Score, SSV = Subjective Shoulder Value.

|  | Suture | Cable | Nothing |
| --- | --- | --- | --- |
| Absolute CS | 0.00 | 0.02 | 0.00 |
| Relative CS | 0.00 | 0.01 | 0.00 |
| SSV | 0.00 | 0.11 | 0.00 |
| CS Pain | 0.00 | 0.00 | 0.01 |
| Flexion | 0.01 | 0.08 | 0.03 |
| Abduction | 0.00 | 0.09 | 0.01 |
| External Rotation | 0.97 | 0.73 | 0.02 |
| Internal Rotation | 0.08 | 0.69 | 0.33 |
| Force | 0.04 | 0.05 | 0.27 |
| Follow-up (months) | 32 +- 15 | 74 +- 15 | 73 +- 14 |
